# Label-free and real-time monitoring of single cell attachment on template-stripped plasmonic nano-holes

**DOI:** 10.1038/s41598-017-11383-x

**Published:** 2017-09-08

**Authors:** Long Tu, Xuzhou Li, Shengtai Bian, Yingting Yu, Junxiang Li, Liang Huang, Peng Liu, Qiong Wu, Wenhui Wang

**Affiliations:** 1 State Key Laboratory of Precision Measurement Technology and Instrument, Department of Precision Instrument, Tsinghua University, Beijing, 100084 China; 2 Department of Biomedical Engineering, School of Medicine, Tsinghua University, Beijing, 100084 China; 3 School of Life Sciences, Tsinghua University, Beijing, 100084 China

## Abstract

Leveraging microfluidics and nano-plasmonics, we present in this paper a new method employing a micro-nano-device that is capable of monitoring the dynamic cell-substrate attachment process at single cell level in real time without labeling. The micro-nano-device essentially has a gold thin film as the substrate perforated with periodic, near-cm^2^-area, template-stripped nano-holes, which generate plasmonic extraordinary optical transmission (EOT) with a high sensitivity to refractive index changes at the metal-dielectric interface. Using this device, we successfully demonstrated label-free and real-time monitoring of the dynamic cell attachment process for single mouse embryonic stem cell (C3H10) and human tumor cell (HeLa) by collecting EOT spectrum data during 3-hour on-chip culture. We further collected the EOT spectral shift data at the start and end points of measurement during 3-hour on-chip culture for 50 C3H10 and 50 HeLa cells, respectively. The experiment results show that the single cell attachment process of both HeLa and C3H10 cells follow the logistic retarded growth model, but with different kinetic parameters. Variations in spectral shift during the same culture period across single cells present new evidence for cell heterogeneity. The micro-nano-device provides a new, label-free, real-time, and sensitive, platform to investigate the cell adhesion kinetics at single cell level.

## Introduction

Cell attachment is the ability of anchorage-dependent cell sticking to and spreading out on another cell or an extracellular matrix (ECM) by its surface chemical bonds and it has fundamental significance in basic research of life sciences^1, 2^. And *in vitro*, cell attachment is studied by the anchorage-dependent attachment between mammalian cells and the substrate. Highly related to cell communication and migration, cell attachment plays an essential role for individual cells in interacting with tissues or organs. The affinity strength between cells and substrate accomplished by cell attachment molecule (CAM) is a very significant determinant event for a series of diseases such as cancer^3^ and atherosclerosis^4^. The process of static *in vitro* cell attachment can be divided into three stages, i) the initial sedimentation of the cell body to its substrate by electrostatic interaction, ii) the following flattening and spreading of cell body on substrate by integrin bonding, and iii) final spreading and stable adhesion by focal adhesion between the cell and its substrate^5^. In order to deeply understand cell attachment mechanism and monitor the dynamic process, a variety of measuring methods have been developed to study related phenomena of attachment, such as polyacylamide-traction force microscopy (PA-TFM) for studying the traction force of single cell, micropatterning for providing microenvironment for single cell studies, and three dimensional traction force quantification (3D-TFM) for the single cell culture and observation, etc^2^. Although each detection technique has its own advantages, none of them can monitor cell attachment in a label-free way and let alone combine two advantages of label-free and real-time together. Other limitations such as low-throughput measurement, high equipment cost and time consumption also seriously constrain the application. A label-free and real-time, user-friendly and low cost single cell attachment detection method is greatly demanded in this field.

Recently, label-free biochemical measurement based on extraordinary optical transmission (EOT) has been proposed and successfully demonstrated in applications such as molecular adsorption and protein-protein binding dynamics for the advantages of simple procedure, low cost and non-invasive^6–17^. The core sensing element of EOT based sensors is a noble metal (gold or silver) thin film perforated with nano-hole arrays. Such periodic sub-wavelength nano-holes result in a change or shift of the EOT transmission spectrum in association with the refractive index change of the medium in the near field of the metallic surface. In practice, the spectral shift can be measured at the spectral peaks and EOT-based biochemical measurement has the paramount advantages of label-free, real-time, simplified optical path, and easy integration with microfluidic channels^18–25^. Therefore, we propose to monitor the cell attachment process by integrating microfluidic channels with the nano-hole-structured substrate. We can monitor the cell attachment process by spectral shift simply because the cell alters its distance and adhesion degree of the substrate, which correlates to the effective refractive index of the medium above the gold thin film. To achieve single-cell measurement, we also design the microfluidic channels to have a matrix of single-cell trapping units so that cells are separated from each other.

At the early stage, periodic sub-wavelength nano-holes on thin noble metal film for producing EOT were fabricated by focused ion beam (FIB) or electron beam lithography (EBL), which is very expensive, time-consuming and hardly applicable for fabricating large-area (e.g., mm to cm scale) nano-holes. However, large-area nano-holes are desirable for biochemical detection^26^. Recently, template-stripping has been successful for low-cost, mass-replication and high-fidelity fabrication of large-area nano-holes^27–37^. In this paper, we successfully fabricated nano-holes by adapting this template-stripping method.

This paper reports a new EOT-based sensing method to monitor the spectral change during the cell attachment flattening and spreading process for single HeLa and C3H10 cells, using a home-made integrated optofluidic chip with the advantage of label-free and real-time monitoring^25, 38–40^. The integrated optofluidic chip is made by combing the single cell capture and culture polydimethylsiloxane (PDMS) micro-channels with the template-stripped large-area thin gold film perforated with nano-holes. The whole chip is placed in a microscopic cell culture system to maintain the right temperature and CO_2_ conditions for cell growth. By processing the signals from a spectrometer mounted on the microscope, the dynamic cell attachment process is monitored. We found that cell attachment process follows the logistic retarded growth model. By analyzing the wavelength shift of 100 single cells for 3 hours, the heterogeneity of single cell attachment is demonstrated and the normal distribution of wavelength shift is discovered. This paper provides a label-free and real-time optofluidics method to monitor single cell attachment kinetics, and opens new opportunities for studying single cells with this micro-nano-hybrid EOT-based sensing platform.

## Experimental

### Experimental setup

**Figure 1:**
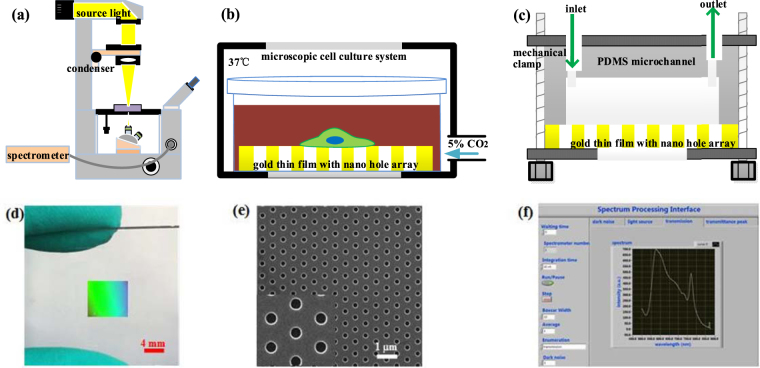
Experimental setup. (**a**) Schematic of the optical microscopy setup and device for spectrum measurement. Note: The light beam is indicated in yellow just for visualization purpose, actually white light is projected on the device. (**b**) Section view of the integrated device used for single cell dynamic attachment measurement. The gold thin film with template-stripped nano-hole array is flat on a petri dish and the whole device is placed in a microscopic cell culture system to maintain the environment of 5% CO_2_ concentration and 37.0 °C temperature for cell culture and attachment. (**c**) Section view of the integrated device used for attachment analysis of many single cells. The PDMS microchannel with single cell capture units and gold thin film perforated with hexagonal nano-hole array are combined with a mechanical clamp. The open injection syringe inlet is 10 mm higher than the outlet to produce gravity pressure difference, which is used to slowly infuse cell culture medium during cell culture. (**d**) Photo of large-area hexagonal nano-hole array on gold thin film with a size of 8 mm × 8 mm. The polychrome color on the gold film is the reflected diffraction light generated by the periodic nano-hole array on gold film. (**e**) SEM of large-area hexagonal nano-hole array (periodicity 600 nm, diameter 180 nm). (**f**) Spectrum processing software graphic interface based on LABVIEW.

The experimental setup is shown in Fig. 1. It is based on the standard Nikon inverted microscope (Ti-U) with microscopic cell culture system, which is used to maintain the environment of 5% CO_2_ concentration and 37.0 °C (within ± 0.1 °C accuracy) temperature for cell culture and attachment. The optical measurement setup (Fig. 1a) is as follows: the filtered and condensed white light radiated from the tungsten-halogen lamp illuminates the nano-hole array from above. Then the light transmitted through the nano-hole array is collected by a 10 × objective and finally is connected to a fiber optic spectrometer (Ocean Optics QE65Pro) via the focal plane of a C-mount side port for spectrum measurement. This setting results in a circular spectrum collection area in a diameter of 5 μm. The optically thick gold film perforated with large-area nano-holes fabricated by template-stripping constitutes the basic sensing unit and cell attachment substrate in experiment. It is used in two configurations. i) The gold film is placed in a petri dish immersed in cell culture medium, with its top surface fully open to the culture medium environment (Fig. 1b). This configuration was adopted initially to develop and demonstrate the constant measurement capability of single cell attachment. ii) The gold film is enclosed by top PDMS microfluidic channels with single cell trap units (Fig. 1c). This configuration was adopted later to trap and culture an array of single cells on-site for collection of data, which corresponded to the start and end points of measurement. Figure 1d shows the picture of a template-stripped nano-holes, and Fig. 1e shows the SEM picture of the nano-hole array. Figure 1f shows the homemade LABVIEW graphic interface for EOT spectrum measurement.

### Data acquisition and processing

Homemade LABVIEW spectrum acquisition and processing software was used (Fig. 1f). For the spectrometer to work well, the integral time of spectrometer was set at 1 s and average frames was set to be 10. The raw spectrum data were processed by the lossless smooth method to remove signal fluctuations and the background spectrum was also removed from the signal spectrum. The resonance peak wavelength was calculated by finding the corresponding wavelength at which the peak light intensity occurred. Finally the spectrum data were exported to MATLAB to obtain the fitting curve and fitting function.

### Fabrication of large-area hexagonal periodic nano-holes

**Figure 2:**
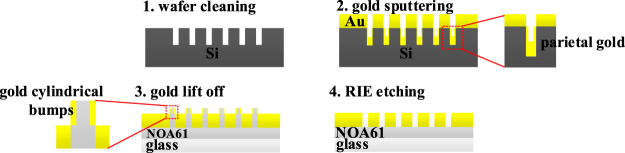
Fabrication process of gold thin film perforated with large-area nano-hole array. (**1**) Wafer cleaning by BOE for 1 min, deionized water 5 min, and nitrogen drying. (**2**) Sputtering gold of 100 nm. (**3**) Gold lift off by NOA61 and transferred on glass slide, the residual NOA 61 and parietal gold film in holes of silicon template turns into gold cylindrical bumps on glass based gold thin film. (**4**) RIE etching with CHF_3_ and O_2_ to etch the residual NOA 61 in holes, and SF_6_ to etch the gold cylindrical bumps.

**Figure 3:**
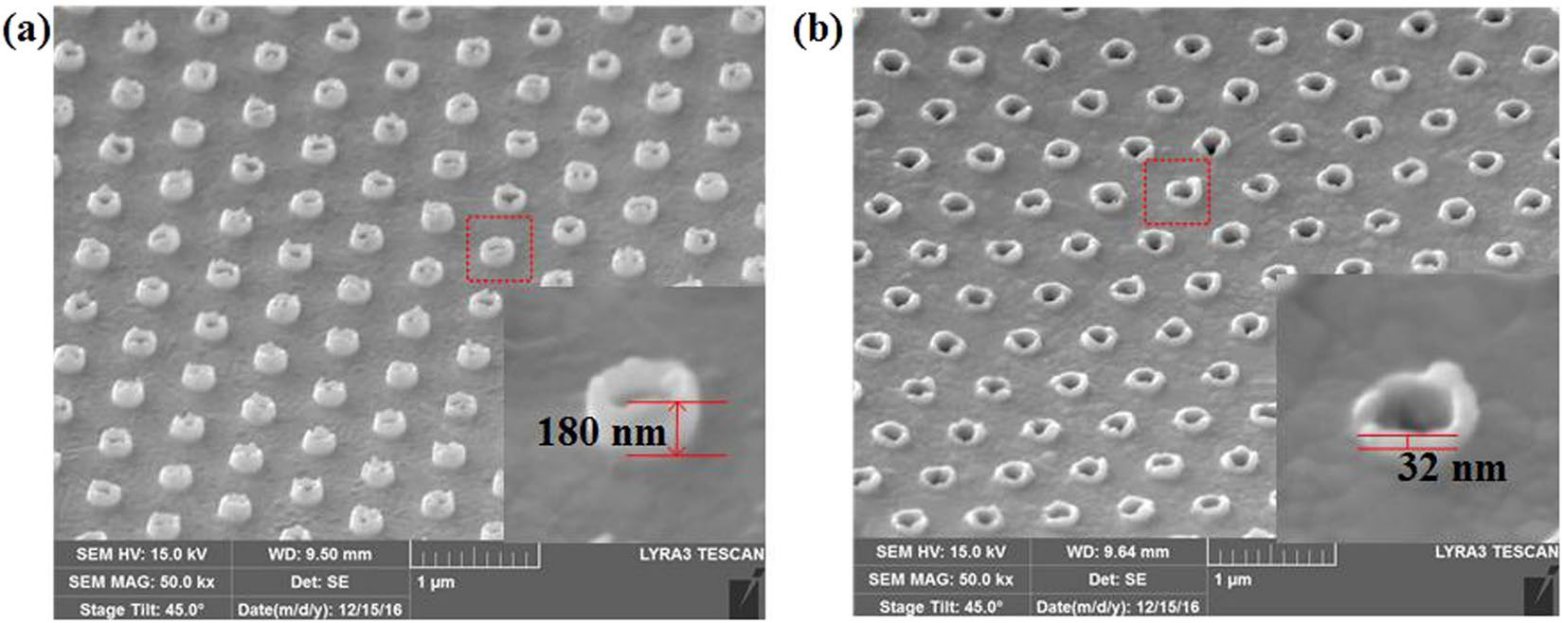
(**a**) SEM of gold thin film with a gold cylindrical bumps with an approximate height of 180 nm attached on each hole. (**b**) SEM of gold thin film with the cylindrical bumps is greatly cut down with an approximately height of 32 nm. The power of SF_6_ RIE etching is 200 W and the pressure is 5 Pa. In order to take pictures of the gold cylindrical bumps, the gold film adhered to glass slide is 45.0° tilted with respect to the electron beam.

**Figure 4:**
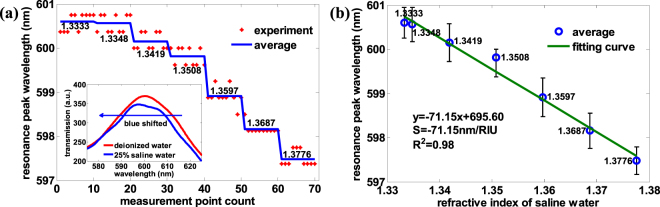
Refractive index sensitivity measurement of gold thin film with many gold cylindrical bumps of an approximate height of 180 nm. (**a**) The resonance peak wavelength with saline water of different concentration (pure water, 1%, 5%, 10%, 15%, 20%, 25%) and the refractive index is 1.3333, 1.3348, 1.3419, 1.3508, 1.3597, 1.3687, 1.3776 respectively. For each concentration of saline water, 10 gathered data is averaged to get the final resonance peak wavelength of the corresponding saline concentration. The inset depicts the transmission spectrum of deionized water and 25% saline water. The peak wavelength around 600 nm is blue-shifted with increased refractive index from deionized water to 25% saline water. (**b**) The refractive index sensitivity fitting line of periodic nano-hole array perforated gold film using saline water, and the refractive index sensitivity is −71.15 nm/RIU with R-square = 0.98. Error bars represent the residual error, which is the difference between measured value and fitted value with the confidence coefficient of 0.95.

**Figure 5:**
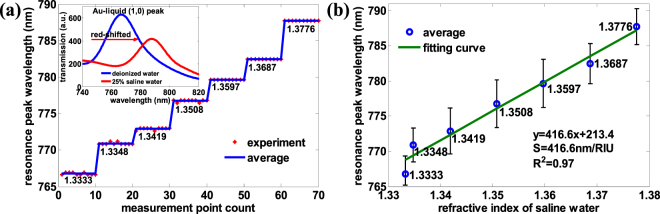
Refractive index sensitivity measurement of gold thin film with gold cylindrical bumps greatly etched by RIE. (**a**) The resonance peak wavelength with saline water of different concentration (pure water, 1%, 5%, 10%, 15%, 20%, 25%). The inset depicts the transmission spectrum of deionized water and 25% saline water. The peak wavelength around 770 nm is red-shifted with increased refractive index from deionized water to 25% saline water. (**b**) The refractive index sensitivity fitting line of periodic nano-hole array perforated gold film using saline water, with the refractive index sensitivity of 416.6 ±1.3 nm/RIU with R-square = 0.97. Error bars represent the residual error, which is the difference between measured value and fitted value with the confidence coefficient of 0.95.

A large-area hexagonal periodic nano-hole Si template with diameter of 180 nm and period of 600 nm (Lightsmyth Corp., USA) was firstly cleaned by a buffered oxide etchant (BOE) solution to remove the remaining SiO_2_ and then rinsed with 1:1 piranha solution and deionized water. After dried by nitrogen, a 100 nm-thick Au film was deposited onto the Si mold by magnetron sputtering machine (JR-2B, Jinsheng Corp., China). The initial 30 nm-thick gold film was deposited at the rate of 40 Å/s and the remaining 70 nm-thick film at 90 Å/s. After gold deposition, a UV-curable optical epoxy (NOA 61, Norland Products) was uniformly covered on the gold surface and compressed by a glass slide, and then cured for 30 min under UV light (100 W) with central wavelength of 365 nm. Because the adhesion of gold on glass by epoxy is greater than on the Si template, the gold thin film with nano-hole array was transferred to the glass substrate when the Si template was peeled off (Fig. 2). Because the epoxy thickness is hard to control, there is always excess epoxy that fills in the holes to be peeled off so that some cylindrical shell with a height of 180 nm stands out from each hole (Fig. 3a). These residual cylindrical bumps seriously decreased the refractive index sensitivity of the large-area hexagonal periodic nano-hole array to 71.15 nm/RIU. In the meanwhile, the resonance peak wavelength was abnormally blue-shifted rather than red-shifted when the refractive index of the medium on the gold thin film increased (Fig. 4). To mitigate the problem, we used reactive ion etching (RIE) of CHF_3_ (28 sccm) and O_2_ (2 sccm) to etch the NOA 61 ultraviolet adhesive in the cylindrical bumps^41^, and then sulfur hexafluoride (SF_6_) to etch the gold cylindrical bumps. After SF_6_ RIE etching for 20 minutes (30 sccm, 5 Pa), the gold cylindrical bumps was greatly cut down (Fig. 3b) and the resonance peak wavelength retained red-shifted with increased refractive index on gold surface. Using saline water of different concentrations (pure water, 1%, 5%, 10%, 15%, 20%, 25%) for validation, the refractive index sensitivity of RIE etching large-area hexagonal periodic nano-hole array gold thin film was obtained with a value of 416.6 ± 1.3 nm/RIU (Fig. 5), which is very close to the sensitivity (~500 nm/RIU) of the FIB fabricated noble metal nano-hole array (e.g., an array size of 30 × 30 or 10 × 10, and period 500 nm)^42–46^. The relationship between concentration w and refractive index of saline water n is taken from literature^47^, where n = 1.333 + 0.1783 w.

Note here the fabrication process was different than mostly-used template-stripping methods in that we added RIE process afterwards to remove the excess epoxy for enhanced RIU sensitivity. This complicates the fabrication process and need to be optimized in order to make it cheap for scaling-up the production.

### Design and fabrication of single cell trap units

Here, we design the single cell trap units by following the passive-flow microfluidic channel, which can be placed in the microscopic cell culture system for real-time observation and maintenance of the normal cell activity, allowing convenient exchange of cell nutrient solution for cell growth without cell dislocation^48^. Schematic diagram of our PDMS microchannel on gold thin film is shown in supporting information Figure S1a and flow simulation results of surface velocity and streamline plots at 100 μm/s inlet flow velocity are demonstrated in Figure S1b. Using standard soft lithography and molding technique (see supporting information), the PDMS microchannel is fabricated. Basically, each trapping unit is composed of two square pillars configured as a V-shaped constriction with the minimum gap of 5 μm (Figure S2). To avoid the interference of the trap units to the spectrum measurement, the trap units have a gap of 2 μm above the gold surface. An array of such constrictions is deployed with deliberately sufficient distance such that each single cell can spread out onto rather an enlarged area in attachment process after trapped.

The bonding between the gold thin film perforated with nano-holes and PDMS microchannel is achieved by a mechanical clamp of two identical ring stainless steel plates (size 25 mm × 25 mm, thickness 0.5 mm) to form a hybrid micro-nano-device. Then two holes were punched through the PDMS layers and hosing-wired outside for fluid in and out. The empty space of the ring plates has a size of 2 mm × 5 mm to allow the light passing through. The commonly used oxygen plasma bonding technique is invalid for gold thin film and thus is not used here. To avoid any liquid leakage through the mechanical clamping, we use gravity to drive fluid with a 10 mm height drop between the inlet medium source and outlet port (Figure S3b)^49^. In this way, the cells are driven gently towards the traps and diffusion can be made possible to culture cells on-site.

### FDTD simulation of spectrum peak shift with the distance between cell and gold thin film

**Figure 6:**
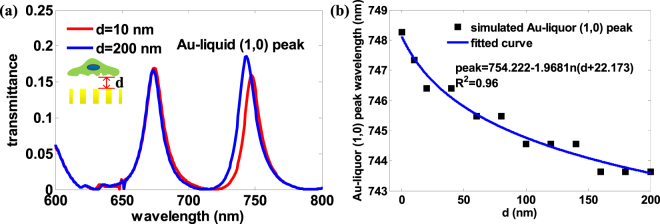
The FDTD simulation results for a simplified model of cell attachment process. (**a**) The transmittance spectrum in association with the cell-substrate distance d. (**b**) Au-liquid (1, 0) resonance peak vs d with R-Square = 0.96. The spectrum peak wavelength is not linear to cell-substrate distance.

The most direct and significant indicator to measure the adhesion of a cell to its substrate is the equivalent distance from the cell bottom to the substrate. The equivalent distance is used here to reflect the irregular cell bottom geometry, which affects the effective refractive index of the medium above the gold surface. According to the EOT theory, the transmission spectrum would be red-shifted when the cell gets closer to the gold thin film, as the refractive index of the cell is bigger than that of cell culture medium. In this paper, to investigate the spectral shift associated with the distance, FDTD Solutions simulation on cell sedimentation process was conducted to simulate the transmittance of gold nano-hole array for a range of distance values. FDTD Solutions is a 3D Maxwell solver which can analyze the interaction of light with wavelength-scale nano-structures. The gold nano-hole array structural parameters in FDTD simulation are set equal to the real fabrication parameters (Fig. 1b) and the simulation model is shown in supporting Information Figure S4. The refractive indexes of cell culture medium and cell are 1.35 and 1.392 respectively^50^. The boundary conditions for x axis and y axis are anti-symmetric and symmetric respectively, and boundary conditions for z axis is perfectly matched layer (PML). The mesh grid is non-uniform and grid step is 5 nm. The transmission spectrum of EOT is usually sensitive to the surface refractive index changes within a distance of 200 nm^51^. To simplify the simulation model, we assumed the cell has a sinusoidal bottom surface when touches upon its substrate and the equivalent distance in simulation is limited within 200 nm. As a large cell is unlikely to descend precisely parallel to the nanohole substrate, there would be geometric fluctuations in cell bottom membrane. Because the adhesion substrate topology is of great significance to adherent cell bottom morphology^52–57^, in this paper, we assume that the adherent cell bottom morphology is adjusted by the nano-hole array in the FDTD simulation model. Thus we use the sinusoidal wave surface model in FDTD structure library. The simulation parameters of sinusoidal wave surface model are set as follows: the thickness is 10 nm to approximately equal to cell membrane thickness, the amplitude is 100 nm to be the same as gold film and hole thickness, the period is 1200 nm to be double of nano-hole array period. Figure 6a shows the spectra for two distances of 10 nm and 200 nm as example. The result confirms that as the distance gets smaller, the Au-liquid (1, 0) resonance peak is red-shifted. We further reinforce this result by plotting the FDTD simulated Au-liquid (1, 0) resonance peak vs distance in Fig. 6b. We found that the relation between Au-liquid (1, 0) peak and the distance d can be fitted well by the logarithm function, i.e., peak = 754.222-1.968 ln (d + 22.173). This implies that the less distance from the cell to gold thin film with nano-hole array, the greater the Au-liquid (1, 0) peak wavelength, and the more red shift for the peak.

### Experiment procedure

To measure the single-cell dynamic attachment process, we used the experiment setup demonstrated in Fig. 1b. In order to comparatively study the attachment of different types of cells, we used HeLa cell and C3H10 cell in the monitoring process. The experiment steps are as follows. Firstly, the cell suspension with cell density of 10^4^ per milliliter was dropped onto the gold thin film perforated nano-hole array placed in the middle of confocal special dish. After 5 minutes, when the cell culture medium settled down, we sought for a cell that had tendency of attachment by recognizing the ovality with the help of a cell biologist. The criterion counted down upon the cell morphology. When the cell looked like an ellipse and not so round, it was highly likely to recover attachment on the substrate. Finally we centered this cell in the field of view of the microscope. Then, in the following 3 hours, we constantly collected the transmission spectrum passing through the cell attached on the gold thin film. The experimental device was thoroughly sterilized by high pressure steam sterilization pot and ultraviolet radiation beforehand.

**Figure 7:**
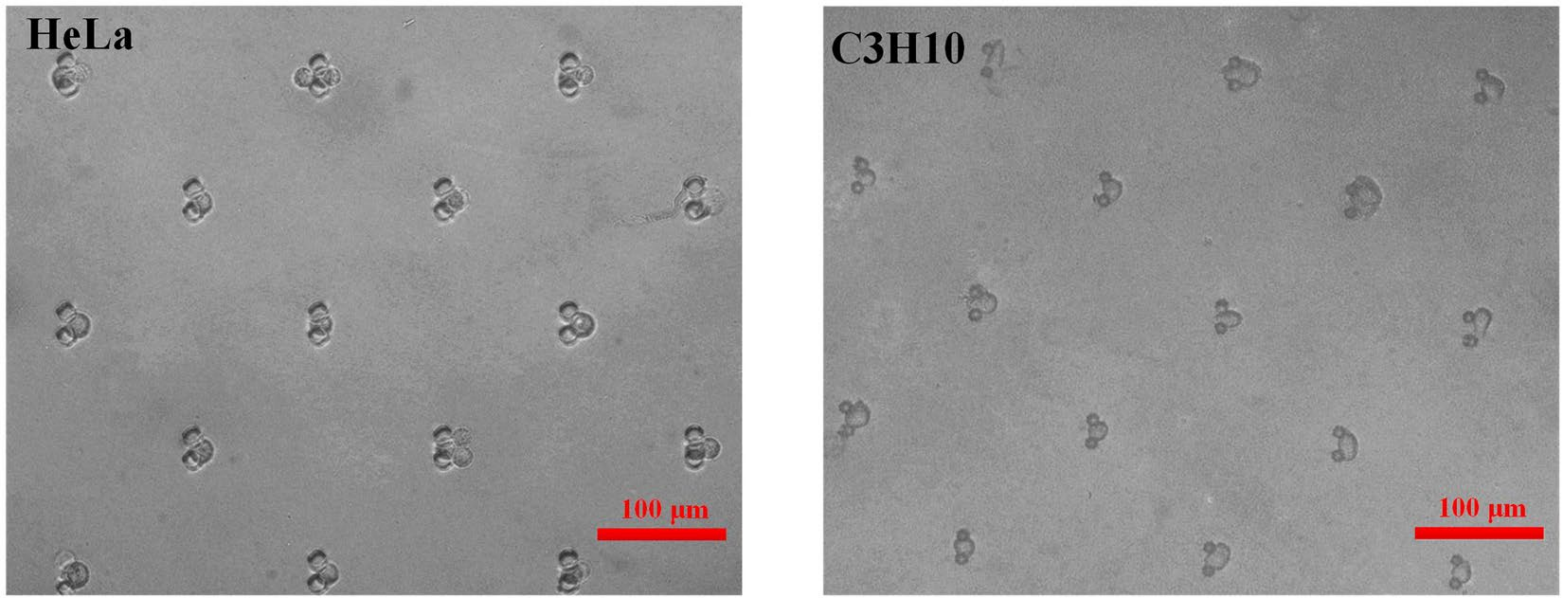
Cell trap efficiency of 100% for HeLa and C3H10 cells by V-shaped constrictions. The cells are immediately trapped when they pass through the constrictions. Most trapping units capture one single cell rather than multiple cells to meet our needs of single-cell measurement.

To collect data reflecting the attachment degree for a number of single cells, we used the experiment setup shown in Fig. 1c to trap and culture an array of single cells on-site. Both HeLa cells and C3H10 cells were measured individually at the start and end points of measurement to make a contrast. The experiment steps are as follows. Cell suspension was dropped into the open injection syringe and the outlet syringe was pulled at a constant and slow speed to produce negative pressure and drive the cell suspension flow from the inlet syringe to outlet syringe (Figure S3a). When the cell suspension flowed through the PDMS microchannel, the cells were trapped at V-shaped constrictions with an efficiency of 100% (Fig. 7). After cell trapping completed, the piston of outlet syringe was pulled out from the outlet syringe to make the outlet syringe be open to atmosphere (Figure S3b). By adjusting the relative height, the open injection syringe inlet is 10 mm higher than the outlet to produce gravity pressure difference driving cell suspension flow with tiny flow rate, which is used to infuse the cell with culture medium. The EOT spectrum during attachment process is obtained by subtracting the spectrum of cell culture medium adjacent to the detected cell (about a distance of cell diameter) from the spectrum of light passing through the detected cell, whose aim is to eliminate the background signal caused by cell culture medium. All the experimental device is sterilized beforehand by high pressure steam sterilization pot and ultraviolet radiation, and the cell suspension with a cell density of 10^5^ per milliliter, and HeLa and C3H10 cells were prepared by general processing method^58^ (supporting information).

## Results

**Figure 8:**
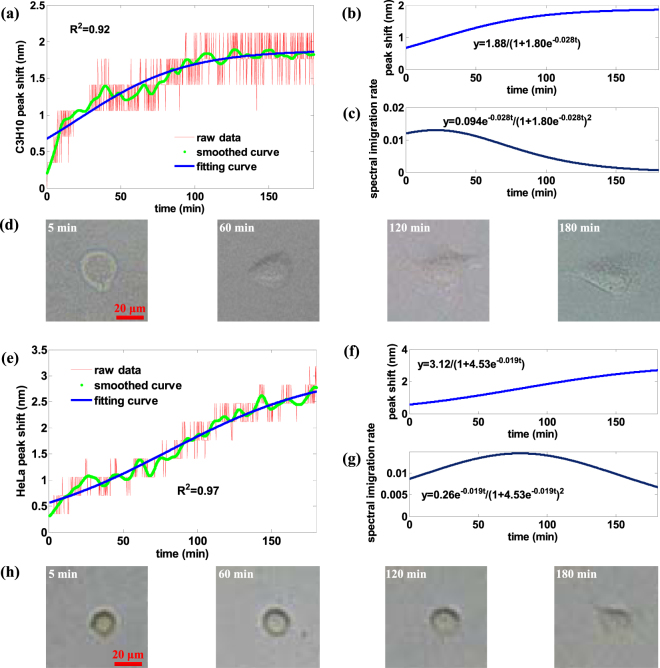
Experimental results of single cell dynamic attachment process monitoring. (**a**) Peak shift raw data, smoothed curve and fitted curve of the single C3H10 cell, with fitted R-square of 0.9218. (**b**) Peak shift smoothed curve and fitted curve of the single C3H10 cell. (**c**) Spectrum peak change rate of the single C3H10 cell. (**d**) The microscopic image of C3H10 cell after 5 minutes, 60 minutes, 120 minutes, 180 minutes of measurement. At 5 minutes, its boundary contour is not so round which indicates its tendency of attachment. (**e**) Peak shift raw data, smoothed curve and fitted curve of the single HeLa cell, with fitted R-square of 0.9732. (**f**) Peak shift smoothed curve and fitted curve of the single HeLa cell. (**g**) Spectrum peak change rate of the single HeLa cell. (**h**) The microscopic image of HeLa cell after 5 minutes, 60 minutes, 120 minutes, 180 minutes of measurement. Compared with the C3H10 cell, the shape of HeLa cell change extent in the same period of attachment time is smaller.

The time-varying transmission spectrum data and morphology images during cell attachment process for both HeLa and C3H10 cells were plotted in Fig. 8a–d and Fig. 8e–h separately, exhibiting an obvious pattern of constant increase in attachment with a tendency of stabilization after some time. After 3 hours of real-time measurement, the cell spreads much flattened compared with the start point of attachment (Fig. 8d,h). Overall, the spectral shift data over time can precisely reflect the cell attachment kinetics.

Due to the resolution of the spectrometer (0.386 nm), the raw data were recorded as stepwise which implies the cell attachment is a dynamic process, indicated by the stair-up-and-down fluctuations in signal. A higher-resolution spectrometer may help reveal more details on what happens during cell attachment. To reflect the signal trend better, the raw data were lossless smoothened to remove the detailed fluctuations. We can see the curve is yet not monotonically increasing - a number of local peaks and valleys exist along the whole curve, which we believe is due in large part to cellular vibrant activity (e.g., migration) in the dynamic attachment process. To further seek for a time-dependent mathematical description of the spectrum peak shift curve, the two lossless smooth curves were fitted by logistic retarded growth model in Fig. 8a and Fig. 8e with R-square of 0.9218 and 0.9732 respectively. The good fitting indicates the logistic retarded growth model sufficiently explains the single-cell dynamic attachment process very well. Though the process could be modeled by other functions, for example, polynomial functions, the logistic retarded growth model was favored because this one-variable function had been used in biology for species number increase prediction. For C3H10 and HeLa cells, their fitting function parameters reveal the difference existing in adhesion dynamics between the two kinds of cells. Compared with HeLa cell, it is much easier for C3H10 cell to recover attachment to gold thin film perforated with nano-hole array, and it is faster for C3H10 cell to achieve attachment stability. Furthermore, we took the first derivative of fitted logistic retarded growth function as the spectral shift rate curve to figure out how quick the cell is supposed to recover attachment on the surface. We found that it took about 25 minutes for C3H10 cell to reach the maximum peak shift rate while about 75 minutes for HeLa cell to do so. After 3 hours, the spectrum peak shift rate of C3H10 cell is nearly 0, which means the attachment process is nearly completed to the equilibrium state. By contrast, the spectrum peak shift rate of HeLa cell is still prominent, which means the attachment process is still proceeding. The whole cell attachment process could be cross-confirmed by the cell morphology microscopic images (Fig. 8d,h).

**Figure 9:**
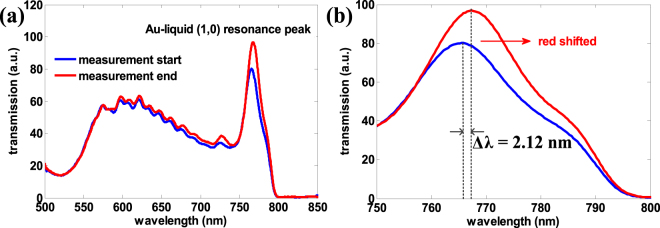
(**a**) The transmission spectrum for C3H10 cell attachment measurement at the start and end points. (**b**) Zoomed-in view of the Au-liquid (1, 0) resonance peak with red shift of Δλ = 2.12 nm.

We compared the simulated EOT spectrum for the simplified cell attachment model (Fig. 6) with the experimental results for the real-time EOT spectrum data of the C3H10 cell at the start and end points of measurement. At the start point of measurement, the peak wavelength 765.3 nm (Fig. 9) obtained in experiment was close to the simulation result, which is 748.3 nm when the cell-substrate distance is 0 nm. Though it is difficult to estimate the real distance in experiment, this close agreement of peak wavelength between the simulation and experiment confirmed in some sense that the cell already finished sedimentation stage and the cell-substrate distance could be less than 200 nm. In experiment, we recorded the spectral shift was only 2.12 nm during the 3-hour measurement process. While the simulation results show that this shift may be 4.6 nm, approximately 2 times of the experimental value when the cell-substrate distance changes from 200 nm to 0 nm. This deviation may imply again that the cell was within 200 nm distance to the substrate when the measurement was started. Overall, the simulation can help describe the cell attachment trend qualitatively, if not quantitatively.

**Figure 10:**
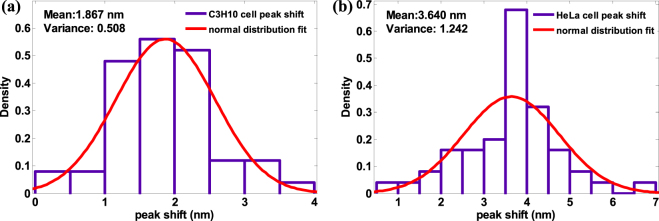
The EOT spectral shift during the 3-hour measurement of cell attachment for two types of cells with 50 cells per type. The spectral shift pattern follows normal distribution. (**a**) C3H10 cells. (**b**) HeLa cells.

For each type of C3H10 and HeLa cells, we obtained the transmission spectrum peak shift between the start and end points of measurement (3 hours and 5 minutes after) for each kind of cells. We found the data for both C3H10 and HeLa cells, as a group, collectively agree with the normal distribution (Fig. 10). It was verified by Kolmogorov-Smirnov test, which is often used to judge whether a set of data-points satisfy normal distribution or not. The results are that for C3H10 cell, the mean and variance is 1.867 nm and 0.508 nm, while for HeLa cell the mean and variance is 3.640 nm and 1.242 nm. The greater the mean of HeLa cell than C3H10 cell indicates the HeLa cell has stronger adhesion ability compared with C3H10 cell at the condition of same attachment time. This result is largely consistent with the biological observation: HeLa cell has much stronger adhesion force than C3H10 cell in the same culture environment and same incubation time, because the trypsin digestion time of adherent HeLa cells (about 5 minutes) is longer than adherent C3H10 cells (about 1 minutes). Since the adhesion strength between cell and substrate is positively related to the number of chemical bonds on the contact surface^59–67^, and the latter is also positively related to the corresponding peak shift, Then we can use the peak shift as a quantitative indicator for the cell-substrate attachment strength. These experiment results demonstrate the cell heterogeneity of the same type of cell in the process of attachment, and discover the relative cell adhesion strength of one group of cell obeys the normal distribution.

## Conclusion

This paper reports on a new method employing a micro-nano-device for label-free and real-time monitoring of single cell attachment on template-stripped plasmonic nano-holes. The device is essentially based on the gold thin film perforated with periodic, near-cm^2^-area, template-stripped nano-holes, which can generate EOT with a high sensitivity (416.6 ±1.3 nm/RIU) in refractive index. The gold nano-hole-structured substrate is configured to work with PDMS microfluidic channels that consist of an array of single cell trap units to allow high-throughput cell attachment measurement at single cell level. Using this device, we successfully demonstrated label-free and real-time monitoring of the dynamic cell attachment process at single cell level for C3H10 stem cell and HeLa cancer cell during 3-hour culture period. We also collected the EOT spectral shift data at the start and end points of measurement for 100 single cells to show its potential in high-throughput application. The experiment results show that the single cell attachment process follows the logistic retarded growth model, but with different kinetic parameters for different cells. The normal-distributed spectral shift recorded during 3-hour measurement for 100 cells provides new evidence for cell heterogeneity in cell attachment process. We envision this new platform would open new research opportunities in single cell attachment studies.

## Electronic supplementary material

Label-free and real-time monitoring of single cell attachment on template-stripped plasmonic nano-holes
