# Supplementary material for: Label-free and real-time monitoring of single cell attachment on template-stripped plasmonic nano-holes
Source: Sci Rep. 2017 Sep 8;7:11020. doi: 10.1038/s41598-017-11383-x (PMC5591264; doi:10.1038/s41598-017-11383-x)
Supplement: Supplementary file 1 — Label-free and real-time monitoring of single cell attachment on template-stripped plasmonic nano-holes [file 41598_2017_11383_MOESM1_ESM.doc]

**Supplementary Information**

**Label-free and real-time monitoring of single cell attachment on template-stripped plasmonic nano-holes**

**Long Tu**^1^**, Xuzhou Li**^1^**, Shengtai Bian**^2^**, Yingting Yu**^3^**, Junxiang Li**^3^**, Liang Huang**^1^**, Peng Liu**^2^**, Qiong Wu**^3^**, and Wenhui Wang**^1^*****

1State Key Laboratory of Precision Measurement Technology and Instrument, Department of Precision Instrument, Tsinghua University, Beijing, 100084, China.

2Department of Biomedical Engineering, School of Medicine, Tsinghua University, Beijing, 100084, China.

3School of Life Sciences, Tsinghua University, Beijing, 100084, China.

*Corresponding author:

Wenhui Wang, PhD, Associate Professor

Email: wwh@tsinghua.edu.cn

**PDMS microchannel fabrication**. Firstly, a seed layer of BN303-30 (Kempur Corp., China) was deposited on a glass slide, including spin coating (500 rpm 10 s, 2000 rpm 35 s, expected height is 30 μm), pre bake (80 ℃ 30 min), UV exposure without a lithography mask (7.8 mw/cm^2^ 34 s), and hard bake (140 ℃ 30 min). Secondly, the SU-8 molding (MicroChem Corp., USA) was formed on seed layer, including spin coating (500 rpm 10 s, 3000 rpm 35 s, measured height is 25 μm), pre bake (65 ℃ 3 min, 95 ℃ 6 min), UV exposure with lithography mask (7.8 mw/cm^2^ 31 s), post-exposure bake (65 ℃ 1 min, 95 ℃ 5 min), development (SU-8 developer), and hard bake (150 ℃ 2 h). Thirdly, the PDMS microchannel was fabricated by molding on SU-8 layer, including PDMS mixed by 10:1 elastomer base and curing agent (Sylgard 184, Dow Corning), degassing (30 min) to remove residual air bubbles, baking (80 ℃ 4 h), PDMS molding peeled off from SU-8 layer. After being punched and cleaned using absolute ethyl alcohol, the PDMS layer was bonded to the gold thin film perforated with nano-hole array with a mechanical clamp.

**HeLa Cell preparation**. HeLa cells were firstly cultured in DMEM supplemented with 10% (v/v) heat inactivated fetal bovine serum in an incubator with constant temperature of 37 ℃. Then the HeLa cells were rinsed with phosphate buffer solution (PBS pH 7.4), and treated with trypsin–EDTA (0.25%) for 5 min and incubated at 37 ℃ for 10 min to be detached from the petri dishes. The cell suspension was then supplemented with the same volume of DMEM with 10% (v/v) serum to stop the reaction of trypsin. After being centrifuged for 5 min at 1000 rpm, the cells were resuspended in 1 mL DMEM supplemented with 2% fetal bovine serum. The cell suspension solution was dripped onto the petri dishes for single cell measurement or open injection syringe for multiple cell capture.

**C3H10 Cell preparation**. Mouse embryonic fibroblast cells C3H10 were cultured in Eagle's Basal medium with 2 mM L-glutamine, 1.5 g/L sodium bicarbonate and Earle's BSS (Hyclone) containing 10% (v/v) fetal bovine serum (FBS)(Gibco), 100 U penicillin ml-1 and 0.1 mg streptomycin ml-1 (Corning). All cells were cultured in humidified conditions with 95 % air and 5 % CO_2_ at 37 ℃. Before the experiment began, 0.05% (w/v) Trypsin- EDTA (Gibco) was used to digest cells. After termination with complete growth medium and centrifugation with 1000 rpm for 5 min, cells were re-suspended in growth medium containing 50% (v/v) Trypsin- EDTA to make the cell density as 10000/ml.


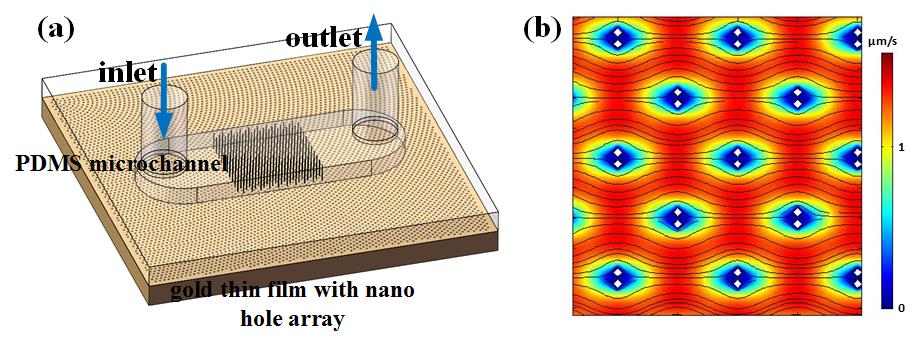


**Figure S1**. (a) [Schematic](javascript:void(0);) [diagram](javascript:void(0);) of the PDMS microchannel on gold thin film perforated with large-area nano-hole array. PDMS channel and gold thin film’s thickness dimensions not to scale. (b) Flow simulation results of surface velocity and streamline plots for 100 μm/s inlet flow velocity.

**Figure S2**. PDMS V-shaped constrictions for single cell trapping. Currently, there are 10 × 10 cell trapping units deployed for demonstration purpose. The array could be up-scaled to a bigger size easily. The height of the trap is 25 μm measured by VEECO DEKTAK 150 profiler.

**Figure S3**. [Schematic](javascript:void(0);) [diagram](javascript:void(0);) for single cell trapping into arrays and on-site culture on the gold nano-hole-structured substrate. (a) Cell suspension was loaded into the open injection syringe and the outlet syringe was pulled at a constant and slow speed to produce [negative](javascript:void(0);) [pressure](javascript:void(0);) and drive the cell suspension flow from inlet syringe to outlet syringe. (b) By adjusting the [relative](javascript:void(0);) [height](javascript:void(0);), the open injection syringe inlet was set 10 mm higher than the outlet to produce gravity pressure difference driving cell suspension flow with [tiny](javascript:void(0);) [flow](javascript:void(0);) [rate](javascript:void(0);), in order to infuse the cell culture medium for on-site cell culture.

**Figure S4.** [Schematic](javascript:void(0);) [diagram](javascript:void(0);) of FDTD simulation model for the spectral shift simulation of the distance between cell and gold thin film. The cell-substrate distance varies from 0 nm to 200 nm with an interval of 10 nm from 0-200 nm. The period of the nano-hole array is 600 nm and the nano-hole diameter is 200 nm. The simulation [parameters](javascript:void(0);) of sinusoidal wave surface model are set as follows: the thickness is 10 nm to approximately equal to cell membrane thickness, the amplitude is 100 nm to be the same as gold film and hole thickness, the period is 1200 nm to be double of nano-hole array period.

**Table 1**. Refractive index sensitivity of large area nano-hole array fabricated by some other groups

| **Research group** | **reference** | **Large area nano-hole fabrication method** | **sensitivity** |
| --- | --- | --- | --- |
| A.-M. Haghiri-Gosnet | 28 | soft UV nanoimprint lithography + lift off | 149.6 nm/RIU |
| Alexandre G. Brolo | 29 | interference lithography + lift off | 271±7 nm/RIU |
| Hooman Mohseni | 30 | lift off | Not mention |
| Grégory Barbillon | 31 | Soft UV Nanoimprint  Lithography | 220 nm/RIU |
| Josu Martinez-Perdiguero | 32 | Nanoimprint Lithography | 126 nm/RIU |
| Reuven Gordon | 33 | Template stripping | Not mention |
| Sang-Hyun Oh | 34 | Template stripping | Not mention |
| Nathan C. Lindquist | 35 | Template stripping | Not mention |
| Hatice Altug | 36 | interference lithography + lift off | 615 nm/RIU |
| Sang-Hyun Oh | 37 | Template stripping | 494 nm/RIU |

**Figure S5**. Relationship between attachment strength and peak wavelength shift. The relationship between cell attachment strength and attachment molecular number is positively correlated and has been proved by much literature [60-68]. The relationship between attachment molecular and effective refractive index on gold thin film within 200 nm is obviously positive correlated with each other, because many research groups have measured molecular binding events according to the biosensing principle of nano-hole array. So it is undoubtedly logical that the cell attachment strength is positively correlated with peak wavelength shift of light passing through nano-hole array perforated on gold thin film. More peak wavelength shift quantity means bigger attachment strength.

**Figure S6**. Transmission spectrum of FIB-fabricated nano-hole array at the distance to view center of (a) 0 μm, (b) 10 μm. We measured the intensity of Au-air (1, 0) peak (around 600 nm) obtained by spectrometer by shifting the FIB-fabricated nano-hole array (40×40 holes, period 500 nm, size 20 μm×20 μm) with respect to the center of field of view of the spectrometer. The intensity of Au-air (1, 0) peak decreases rapidly as the nano-holes get further away from the view center. Since the full width at half maximum (FWMH) is about 5 μm, we conclude the spectrum collection area is a circular region with diameter of 5 μm.
